# Correction: A systems biology approach unveils different gene expression control mechanisms governing the immune response genetic program in peripheral blood mononuclear cells exposed to SARS-CoV-2

**DOI:** 10.1371/journal.pone.0320910

**Published:** 2025-03-25

**Authors:** Damariz Marin, Geysson Javier Fernandez, Juan C. Hernandez, Natalia Taborda

There is an error in the corresponding author’s email address. Natalia Taborda’s correct email address is natalia.taborda@uniremington.edu.co.
