# Supplementary figures and images for: A systems biology approach unveils different gene expression control mechanisms governing the immune response genetic program in peripheral blood mononuclear cells exposed to SARS-CoV-2
Source: PLoS One. 2024 Dec 5;19(12):e0314754. doi: 10.1371/journal.pone.0314754 (PMC11620636; doi:10.1371/journal.pone.0314754)

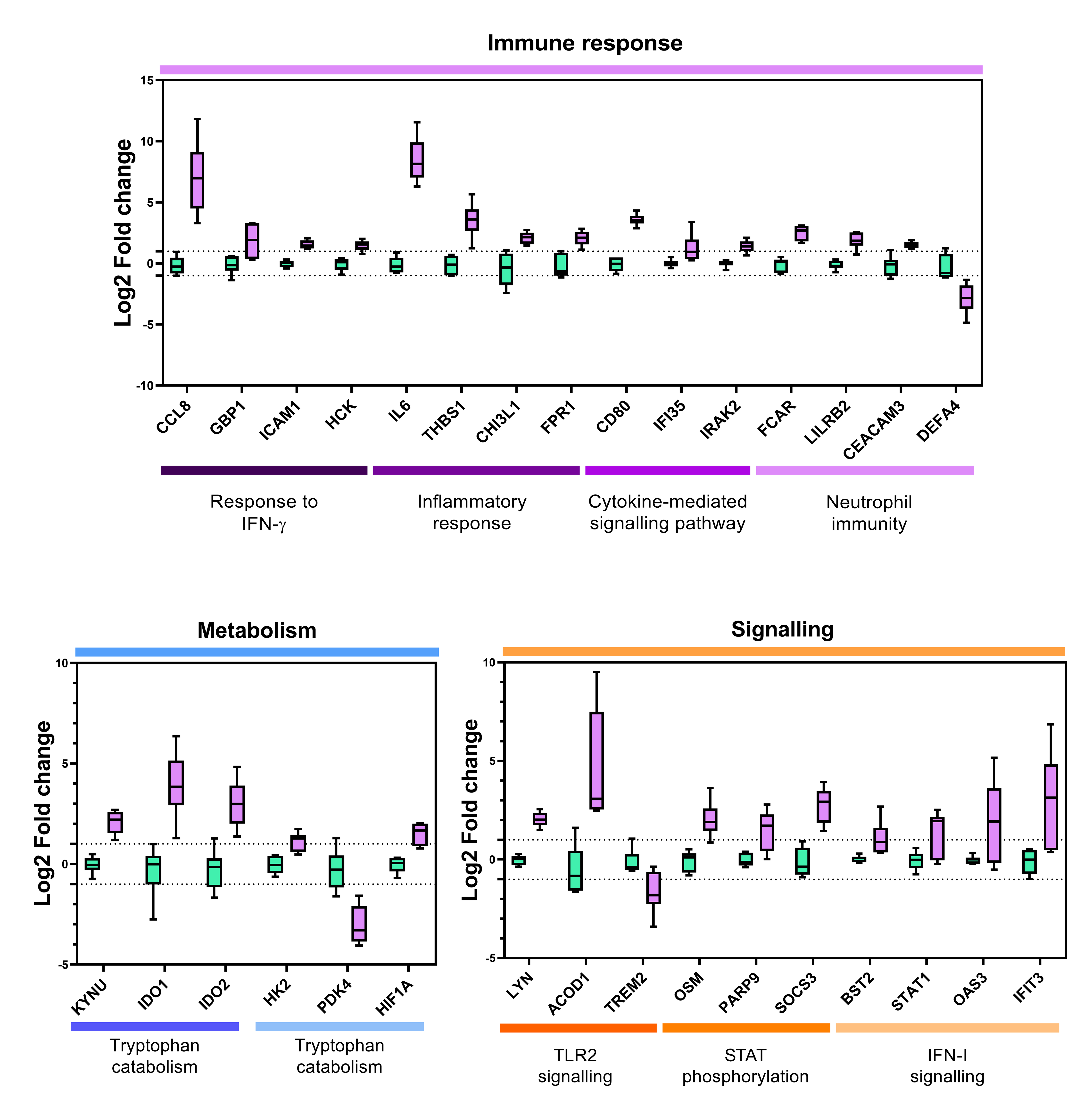

Supplement: S1 Fig — Barplot of DEG selected for each term, to show regulated genes in PBMCs exposed to SARS-CoV-2 represented as log2-fold change (y-axis). (TIF) [file pone.0314754.s001.tif]

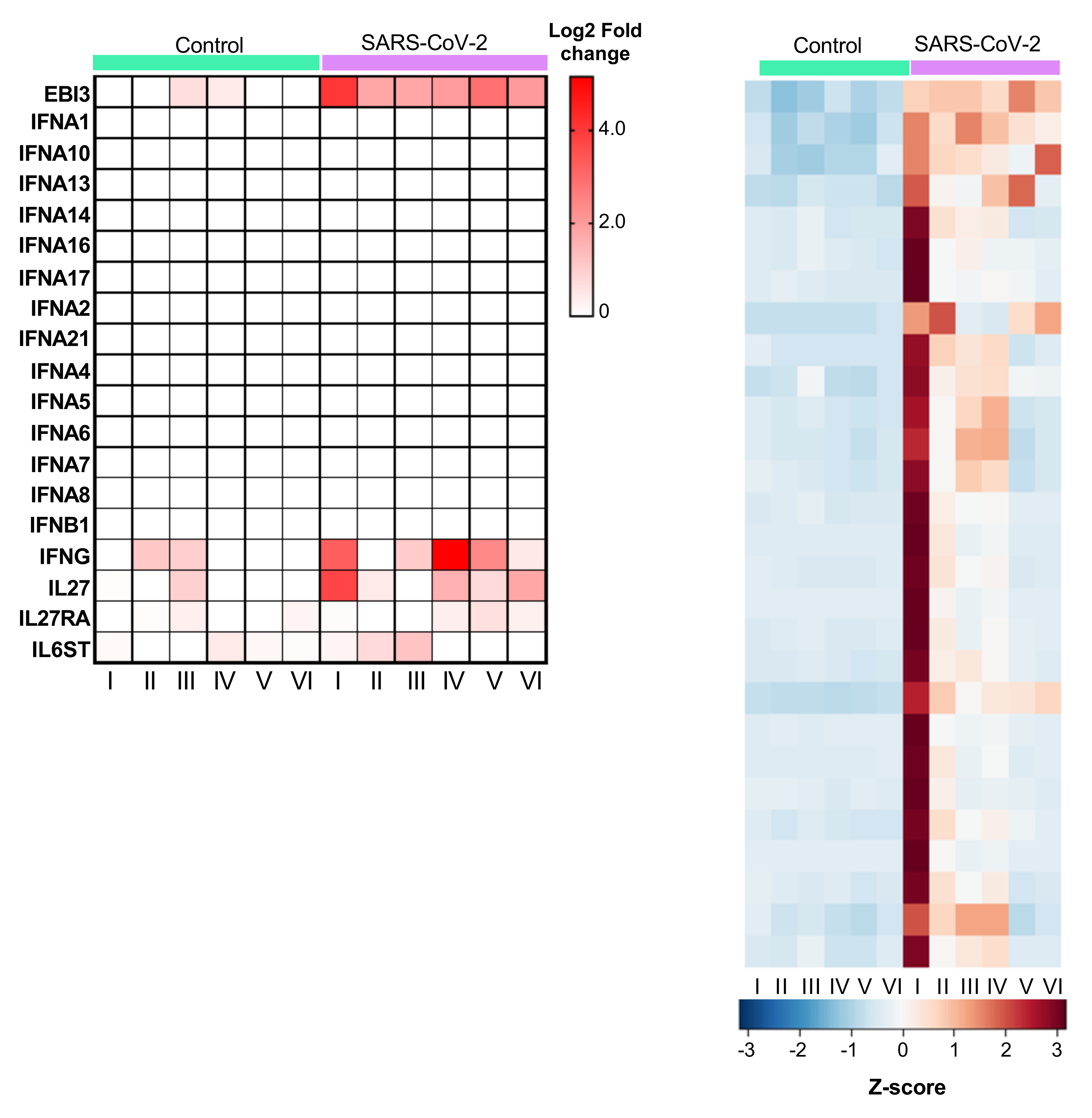

Supplement: S2 Fig — (a) Heatmap of Z-score normalized differentially expressed interferon-related genes of control and SARS-CoV-2-exposed cells. (b) Heatmap of Z-score normalized differentially expressed genes (DEG) of control and SARS-CoV-2-exposed cells. (TIF) [file pone.0314754.s002.tif]
